# Supplementary material for: Unveiling the Bioactive Potential of the Invasive Jellyfish Phyllorhiza punctata Through Integrative Transcriptomic and Proteomic Analyses
Source: Biomolecules. 2025 Aug 4;15(8):1121. doi: 10.3390/biom15081121 (PMC12383608; doi:10.3390/biom15081121)
Supplement: Supplementary file 1 [file biomolecules-15-01121-s001.zip › Suplementary Material/FileS3/PEPFOLD4/XP_065056015_15028/clusters.html]

 PEP-FOLD clusters


Download archive of all models  here   

|  |  |  |
| --- | --- | --- |
| # | file | sOPEP |
| # cluster 1 - Size: 99 |  |  |
|  | XP\_065056015\_15028-model1 | -20.9601 |
|  | XP\_065056015\_15028-model1.1 | -20.933 |
|  | XP\_065056015\_15028-model1.2 | -20.933 |
|  | XP\_065056015\_15028-model1.3 | -20.904 |
|  | XP\_065056015\_15028-model1.4 | -20.904 |
|  | XP\_065056015\_15028-model1.5 | -20.8956 |
|  | XP\_065056015\_15028-model1.6 | -20.8664 |
|  | XP\_065056015\_15028-model1.7 | -20.8163 |
|  | XP\_065056015\_15028-model1.8 | -20.8139 |
|  | XP\_065056015\_15028-model1.9 | -20.8139 |
|  | XP\_065056015\_15028-model1.10 | -20.8139 |
|  | XP\_065056015\_15028-model1.11 | -20.8139 |
|  | XP\_065056015\_15028-model1.12 | -20.8139 |
|  | XP\_065056015\_15028-model1.13 | -20.8007 |
|  | XP\_065056015\_15028-model1.14 | -20.7918 |
|  | XP\_065056015\_15028-model1.15 | -20.784 |
|  | XP\_065056015\_15028-model1.16 | -20.784 |
|  | XP\_065056015\_15028-model1.17 | -20.761 |
|  | XP\_065056015\_15028-model1.18 | -20.7576 |
|  | XP\_065056015\_15028-model1.19 | -20.74 |
|  | XP\_065056015\_15028-model1.20 | -20.7274 |
|  | XP\_065056015\_15028-model1.21 | -20.7126 |
|  | XP\_065056015\_15028-model1.22 | -20.7103 |
|  | XP\_065056015\_15028-model1.23 | -20.7081 |
|  | XP\_065056015\_15028-model1.24 | -20.7058 |
|  | XP\_065056015\_15028-model1.25 | -20.7058 |
|  | XP\_065056015\_15028-model1.26 | -20.7018 |
|  | XP\_065056015\_15028-model1.27 | -20.6981 |
|  | XP\_065056015\_15028-model1.28 | -20.695 |
|  | XP\_065056015\_15028-model1.29 | -20.6946 |
|  | XP\_065056015\_15028-model1.30 | -20.6865 |
|  | XP\_065056015\_15028-model1.31 | -20.6792 |
|  | XP\_065056015\_15028-model1.32 | -20.6755 |
|  | XP\_065056015\_15028-model1.33 | -20.6685 |
|  | XP\_065056015\_15028-model1.34 | -20.6666 |
|  | XP\_065056015\_15028-model1.35 | -20.6624 |
|  | XP\_065056015\_15028-model1.36 | -20.6616 |
|  | XP\_065056015\_15028-model1.37 | -20.6603 |
|  | XP\_065056015\_15028-model1.38 | -20.6548 |
|  | XP\_065056015\_15028-model1.39 | -20.6548 |
|  | XP\_065056015\_15028-model1.40 | -20.642 |
|  | XP\_065056015\_15028-model1.41 | -20.6387 |
|  | XP\_065056015\_15028-model1.42 | -20.6387 |
|  | XP\_065056015\_15028-model1.43 | -20.6294 |
|  | XP\_065056015\_15028-model1.44 | -20.6211 |
|  | XP\_065056015\_15028-model1.45 | -20.6211 |
|  | XP\_065056015\_15028-model1.46 | -20.6211 |
|  | XP\_065056015\_15028-model1.47 | -20.6199 |
|  | XP\_065056015\_15028-model1.48 | -20.6196 |
|  | XP\_065056015\_15028-model1.49 | -20.6184 |
|  | XP\_065056015\_15028-model1.50 | -20.6148 |
|  | XP\_065056015\_15028-model1.51 | -20.6148 |
|  | XP\_065056015\_15028-model1.52 | -20.6148 |
|  | XP\_065056015\_15028-model1.53 | -20.6063 |
|  | XP\_065056015\_15028-model1.54 | -20.605 |
|  | XP\_065056015\_15028-model1.55 | -20.5963 |
|  | XP\_065056015\_15028-model1.56 | -20.5949 |
|  | XP\_065056015\_15028-model1.57 | -20.579 |
|  | XP\_065056015\_15028-model1.58 | -20.5785 |
|  | XP\_065056015\_15028-model1.59 | -20.5765 |
|  | XP\_065056015\_15028-model1.60 | -20.575 |
|  | XP\_065056015\_15028-model1.61 | -20.5707 |
|  | XP\_065056015\_15028-model1.62 | -20.5697 |
|  | XP\_065056015\_15028-model1.63 | -20.5697 |
|  | XP\_065056015\_15028-model1.64 | -20.5679 |
|  | XP\_065056015\_15028-model1.65 | -20.564 |
|  | XP\_065056015\_15028-model1.66 | -20.5619 |
|  | XP\_065056015\_15028-model1.67 | -20.5589 |
|  | XP\_065056015\_15028-model1.68 | -20.5561 |
|  | XP\_065056015\_15028-model1.69 | -20.5543 |
|  | XP\_065056015\_15028-model1.70 | -20.5481 |
|  | XP\_065056015\_15028-model1.71 | -20.5407 |
|  | XP\_065056015\_15028-model1.72 | -20.5341 |
|  | XP\_065056015\_15028-model1.73 | -20.5338 |
|  | XP\_065056015\_15028-model1.74 | -20.5334 |
|  | XP\_065056015\_15028-model1.75 | -20.5297 |
|  | XP\_065056015\_15028-model1.76 | -20.5297 |
|  | XP\_065056015\_15028-model1.77 | -20.5297 |
|  | XP\_065056015\_15028-model1.78 | -20.5196 |
|  | XP\_065056015\_15028-model1.79 | -20.5125 |
|  | XP\_065056015\_15028-model1.80 | -20.5118 |
|  | XP\_065056015\_15028-model1.81 | -20.5096 |
|  | XP\_065056015\_15028-model1.82 | -20.5064 |
|  | XP\_065056015\_15028-model1.83 | -20.5055 |
|  | XP\_065056015\_15028-model1.84 | -20.5041 |
|  | XP\_065056015\_15028-model1.85 | -20.5038 |
|  | XP\_065056015\_15028-model1.86 | -20.4949 |
|  | XP\_065056015\_15028-model1.87 | -20.4921 |
|  | XP\_065056015\_15028-model1.88 | -20.4846 |
|  | XP\_065056015\_15028-model1.89 | -20.4837 |
|  | XP\_065056015\_15028-model1.90 | -20.4771 |
|  | XP\_065056015\_15028-model1.91 | -20.4767 |
|  | XP\_065056015\_15028-model1.92 | -20.4444 |
|  | XP\_065056015\_15028-model1.93 | -20.4443 |
|  | XP\_065056015\_15028-model1.94 | -20.4388 |
|  | XP\_065056015\_15028-model1.95 | -20.4265 |
|  | XP\_065056015\_15028-model1.96 | -20.3869 |
|  | XP\_065056015\_15028-model1.97 | -20.343 |
|  | XP\_065056015\_15028-model1.98 | -20.3282 |
| # cluster 2 - Size: 1 |  |  |
|  | XP\_065056015\_15028-model2 | -18.0673 |
|  |  |  |
